# Protecting the clean athlete, protecting health

**DOI:** 10.1186/2052-1847-7-S1-O2

**Published:** 2015-08-11

**Authors:** Richard Budgett

**Affiliations:** 1 International Olympic Committee Medical and Scientific Director

## 

The vision of the IOC is to change the world for the better through sport and to promote social change. Protecting the health of the athlete is an important part of this vision, in order to protect the integrity of sport, protect elite athletes, protect the health of all those exercising and to promote physical activity. The protection of clean athletes through doping control is also important for the protection of the integrity of sport and of athlete health. The focus of the IOC Medical Commission is the prevention of injury and illness; this is done through world conferences, injury and illness surveillance, International Federations (IF) and National Olympic Committees.

Rowing is an excellent example of a non-contact sport, where overuse injuries are the main problem. Illness and overtraining are also important. Individual physiology and training are crucial to develop the combination of power and endurance needed to compete. Rowing is a perfect sport for the multidisciplinary team of sport and exercise medicine, physiotherapy, physiology, psychology, nutrition and biomechanics specialists.

Priority for the IOC is surveillance and audit, so that changes to rules, to equipment, to training and to technique can be evidence based and the effect on prevention measured. It is important for different sport federations to take every opportunity to work with each other for cross fertilization of best practice and new ideas, including at the IOC IF meetings.

The IOC and world rowing share the priority of protecting health and protecting clean athletes.
